# Optical Imaging Technologies and Clinical Applications in Gastrointestinal Endoscopy

**DOI:** 10.3390/diagnostics15202625

**Published:** 2025-10-17

**Authors:** Khyati Bidani, Vishali Moond, Madhvi Nagar, Arkady Broder, Nirav Thosani

**Affiliations:** 1 Department of Internal Medicine, Saint Peter’s University Hospital/Robert Wood Johnson Medical School, New Brunswick, NJ 08901, USA; khyatibidani@gmail.com; 2 Department of Gastroenterology & Hepatology, West Virginia University, Morgantown, WV 26506, USA; 3 Department of Internal Medicine, Government Medical College, Jamnagar 361006, India; 4 Division of Gastroenterology, Saint Peter’s University Hospital/Robert Wood Johnson Medical School, New Brunswick, NJ 08901, USA; 5 Center for Interventional Gastroenterology at UTHealth (iGUT), Department of Surgery, Division of Endoluminal Surgery and Interventional Gastroenterology, McGovern Medical School at UTHealth, Houston, TX 77030, USA

**Keywords:** gastrointestinal endoscopy, optical imaging, image-enhanced endoscopy, optical coherence tomography, artificial intelligence

## Abstract

Optical imaging technologies expand gastrointestinal endoscopy beyond white-light endoscopy (WLE), improving visualization of mucosal, vascular, and subsurface features. They are applied to the detection of neoplastic and premalignant lesions, inflammatory diseases, and small bowel and pancreatic disorders, though their validation and readiness for routine practice vary. This review critically evaluates both guideline-endorsed and investigational optical imaging techniques across major gastrointestinal indications, highlighting diagnostic performance, level of validation, current guideline recommendations, and practical challenges to adoption. In Barrett’s esophagus, narrow-band imaging (NBI) is guideline-endorsed, while acetic acid chromoendoscopy is validated in expert centers. For gastric intestinal metaplasia and early gastric cancer, magnifying NBI achieves diagnostic accuracies exceeding 90% and is guideline-recommended, with acetic acid chromoendoscopy aiding in margin delineation. In inflammatory bowel disease, dye-spray chromoendoscopy is the reference standard for dysplasia surveillance, with virtual methods such as NBI, FICE, and i-SCAN serving as practical alternatives when dye application is not feasible. In the colorectum, NBI supports validated optical diagnosis strategies (resect-and-discard, diagnose-and-leave), while dye-based chromoendoscopy improves detection of flat and serrated lesions. Capsule endoscopy remains the standard for small bowel evaluation of bleeding, Crohn’s disease, and tumors, with virtual enhancement, intelligent chromo capsule endoscopy, and AI-assisted interpretation emerging as promising adjuncts. Pancreaticobiliary applications of optical imaging are also advancing, though current evidence is still preliminary. Investigational modalities including confocal laser endomicroscopy, optical coherence tomography, autofluorescence, Raman spectroscopy, and fluorescence molecular imaging show potential but remain largely restricted to research or expert settings. Guideline-backed modalities such as NBI and dye-based chromoendoscopy are established for clinical practice and supported by robust evidence, whereas advanced techniques remain investigational. Future directions will rely on broader validation, integration of artificial intelligence, and adoption of molecularly targeted probes and next-generation capsule technologies, which together may enhance accuracy, efficiency, and standardization in gastrointestinal endoscopy.

## 1. Introduction

Gastrointestinal (GI) endoscopy has transformed modern practice by enabling direct visualization, diagnosis, and therapy across the digestive tract. For decades, white-light endoscopy (WLE) has been the standard imaging technique, illuminating mucosa with broad-spectrum visible light to generate natural-color images. While foundational, WLE has notable limitations, as subtle, flat, or early-stage neoplastic lesions may be overlooked, and surface morphology alone provides limited information about subsurface or cellular changes [1]. These diagnostic gaps have driven the development of advanced optical imaging technologies designed to improve detection, characterization, and staging of GI disease.

Fundamentally, these technologies exploit the interaction of light with tissue through absorption, scattering, and fluorescence to generate contrast beyond what WLE can achieve [1,2]. They encompass spectral image-enhancement techniques (e.g., NBI, FICE, i-SCAN), fluorescence-based approaches (autofluorescence imaging, molecular probes), high-resolution or depth-resolved imaging (CLE, OCT), and more experimental methods such as Raman spectroscopy, hyperspectral imaging, photoacoustic imaging, and multiphoton microscopy [1,2,3,4,5].

Although these innovations expand diagnostic possibilities, their validation, adoption, and clinical utility vary widely. Some modalities, such as NBI and dye-spray chromoendoscopy, are supported by randomized controlled trials and incorporated into international guidelines [6]. Others remain investigational, hindered by limited accuracy, high cost, or workflow barriers [7,8].

The novelty of this review lies in its clinically oriented, indication-based synthesis of optical imaging, highlighting not only diagnostic performance but also readiness for clinical practice. We focus on clarifying which technologies are guideline-endorsed and ready for routine care, how comparative evidence informs their diagnostic accuracy and cost-effectiveness, and where emerging modalities currently stand along with the barriers that limit their translation.

To address these goals, we first provide a brief overview of the fundamental principles of optical imaging. We then structure the review by clinical indication: Barrett’s esophagus, gastric premalignancy and early gastric cancer, inflammatory bowel disease, colorectal neoplasia, small bowel disorders, and pancreaticobiliary disease. Each section summarizes the evidence base, guideline status, and clinical takeaways. We also provide comparative summary tables, highlight barriers to adoption, and outline future perspectives, including multimodal integration and artificial intelligence.

By framing optical imaging through the lens of clinical readiness, this review provides gastroenterologists, trainees, and researchers with a clear, evidence-based perspective on current practice and evolving frontiers.

## 2. Methods

Although this is a narrative rather than systematic review, we performed a structured literature search using PubMed, Scopus, and Embase through March 2025, focusing on randomized controlled trials, meta-analyses, guideline statements, and key translational studies. Search terms included but were not limited to “optical imaging endoscopy,” “narrow-band imaging,” “confocal laser endomicroscopy,” “optical coherence tomography,” “fluorescence endoscopy,” “Raman spectroscopy,” “photoacoustic imaging,” and “gastrointestinal neoplasia/IBD.” Additional references were identified from bibliographies of major reviews. To enhance clinical relevance, we prioritized evidence stratified by clinical indication.

## 3. Fundamentals and Classification of Optical Imaging Technology

The performance of optical imaging technologies in gastrointestinal (GI) endoscopy relies on fundamental principles of light–tissue interaction: absorption, scattering, reflection, and fluorescence. By selectively manipulating these properties, different modalities enhance contrast and provide information beyond what is visible with white-light endoscopy (WLE). While WLE remains the clinical baseline, its limited sensitivity for subtle or flat lesions underscores the need for advanced imaging [1,9,10].

To simplify a heterogeneous field, optical imaging modalities can be grouped into the following broad categories (Table 1).

### 3.1. White-Light Endoscopy (WLE)

White-light endoscopy (WLE) remains the universal baseline in GI imaging, producing natural-color images under broad-spectrum illumination. High-definition WLE is now standard in clinical practice. It is widely available, reliable, and effective for routine diagnosis, surveillance, and therapeutic guidance. Its advantages include broad accessibility, ease of use, and compatibility with therapeutic interventions, making it indispensable in daily clinical practice. Nevertheless, its reliance on surface morphology limits sensitivity for flat or subtle lesions, provides no subsurface or cellular-level information, and necessitates random biopsy protocols in conditions such as Barrett’s esophagus and IBD [1,9,10]. These limitations have driven the development of advanced optical imaging technologies aimed at improving detection, characterization, and staging.

### 3.2. Spectral Image-Enhanced Endoscopy

These modalities alter the spectral properties of light, either at the source or through digital reconstruction, to highlight mucosal and vascular details.

- **Narrow-Band Imaging (NBI, Olympus):** Uses optical filters at 415 nm (blue) and 540 nm (green), wavelengths strongly absorbed by hemoglobin, rendering superficial capillaries brown and deeper vessels cyan [11,12]. NBI enhances mucosal and vascular contrast, improves detection of early neoplasia in Barrett’s esophagus, facilitates real-time polyp characterization via the NICE classification, and increases recognition of early gastric cancer [12,13,14]. It is guideline-endorsed by the ESGE for diminutive polyp characterization and by the ASGE for targeted Barrett’s surveillance [15,16]. Advantages include ease of use, no dyes, and robust RCT validation, though limitations include darker images, reduced performance with bleeding or inflammation, and interobserver variability despite standardized classifications [17].
- **FICE (Fuji Intelligent Color Enhancement, Fujifilm):** A digital spectral estimation algorithm reconstructs images at selected wavelengths from standard white-light endoscopy, improving mucosal and vascular contrast. It is used mainly for colorectal polyp characterization, Barrett’s surveillance, and gastric lesion assessment [18]. Flexible presets are available, but validation is less robust than for NBI. Reported sensitivity and specificity for adenoma detection reach 85–92% and 83–90%, though the 2019 ESGE guidelines cite weaker evidence and limited adoption [15,19].
- **i-SCAN (Pentax):** A digital post-processing technology with three modes, surface enhancement, contrast enhancement, and tone enhancement. It is used for Barrett’s esophagus, colorectal polyp differentiation, and assessment of inflammatory bowel disease activity [18]. Its flexibility allows tailored imaging, and studies suggest correlation with histologic inflammation, but evidence remains modest, guideline endorsement is lacking, and adoption is limited by variable settings and a steeper learning curve [20].

### 3.3. Fluorescence-Based Imaging

Fluorescence-based imaging distinguishes normal from abnormal mucosa by detecting natural or introduced fluorophores.

- **Autofluorescence Imaging (AFI):** Excites endogenous fluorophores (collagen, NADH, flavins), displaying normal mucosa as green and dysplasia/neoplasia as magenta [21]. AFI has been tested in Barrett’s esophagus, ulcerative colitis, and early gastric cancer, but adds only ~2% incremental yield over standard endoscopy and shows false-positive rates of 70–80% [22]. Its low specificity and artifact susceptibility have led to a sharp decline in clinical use, though it may serve as an adjunct in multimodal systems [23].
- **Fluorescence Molecular Imaging (FMI):** Employs exogenous probes (e.g., VEGF-A targeted tracers) that bind selectively to neoplastic tissue, enabling high target-to-background contrast [24]. Early trials show feasibility in colorectal, esophageal, and gastric neoplasia, with potential for precision-guided biopsies. However, FMI remains investigational, limited by regulatory hurdles, probe safety, and cost [25,26].

In summary, AFI is largely obsolete in routine practice, while FMI holds promise but awaits clinical translation.

### 3.4. High-Resolution Microscopy

These technologies provide microscopic visualization of cellular structures in vivo, approaching the resolution of conventional histopathology.

- **Confocal Laser Endomicroscopy (CLE):** CLE uses laser excitation with a confocal aperture to generate high-resolution cellular images (1–5 μm), typically enhanced with intravenous fluorescein [27]. Two systems exist: endoscope-integrated CLE (Pentax) and probe-based CLE (Cellvizio, Mauna Kea). RCTs support its role in Barrett’s esophagus, gastric intestinal metaplasia, and IBD, where it improves dysplasia detection and may reduce random biopsies [28,29,30,31]. However, adoption is limited by cost, training, need for contrast, and technical constraints such as narrow field of view and shallow penetration depth (50–250 μm). Thus, CLE remains largely confined to expert centers [31].
- **Endocytoscopy:** Endocytoscopy provides ultra-high magnification (up to 1125×) of mucosal epithelium using topical stains (methylene blue or toluidine blue) [32,33]. It enables nuclear-level visualization with diagnostic accuracy approaching histology; a multicenter study reported 96.9% sensitivity and 100% specificity for colorectal neoplasia [34]. Additional applications include Barrett’s dysplasia and *H. pylori* assessment. Despite promise, adoption is limited outside Japan due to the need for meticulous preparation, dye application, and its very small field of view [32,33,34,35].

Overall, CLE and endocytoscopy highlight the potential of in vivo histology but remain restricted to specialized centers due to cost, workflow, and technical demands.

### 3.5. Depth-Resolved Imaging

Depth-resolved imaging extends visualization beneath the mucosal surface, enabling cross-sectional assessment of tissue microstructure in real time.

- **Optical Coherence Tomography (OCT):** OCT uses low-coherence interferometry with near-infrared light to generate high-resolution cross-sectional images (7–10 μm, depth 1–2 mm) [36,37,38]. It has been applied in Barrett’s esophagus (including volumetric laser endomicroscopy, VLE), early esophageal and gastric cancer, IBD, and pancreaticobiliary strictures [37,38,39,40]. Newer applications include assessment of subepithelial lesions and guidance during endoscopic submucosal dissection [39]. OCT enables wide-field, contrast-free imaging useful for staging and guiding therapy, but clinical use remains limited by high equipment cost, probe-based workflow requirements, need for expert interpretation, and underdeveloped reimbursement models [38]. Consequently, while OCT is clinically valuable in select centers, its broader use remains niche.

### 3.6. Emerging and Experimental Techniques

Several novel optical imaging techniques are in early translational stages. They offer unique diagnostic potential but remain experimental due to technical, regulatory, and workflow barriers.

- **Raman Spectroscopy:** Analyzes inelastic scattering of light to generate molecular “fingerprints” of tissue. Fiber-optic probes have shown high accuracy for early gastric cancer and colorectal neoplasia, with sensitivities around 90% and specificities up to 100% in pilot studies. It also shows potential in blood-based diagnostics and IBD differentiation. Raman spectroscopy offers label-free, highly specific biochemical analysis capable of detecting molecular changes that precede morphological alterations. Despite its promise, clinical use is limited by weak signal intensity, background fluorescence, and difficulty integrating systems into routine endoscopy [41,42,43].
- **Photoacoustic Imaging (PAI):** Combines optical excitation with ultrasound detection, allowing deeper imaging (~2 cm) and functional assessment of vascularity and oxygenation. Early work has demonstrated feasibility in colorectal cancer and inflammatory models, with multimodal systems integrating PAI, ultrasound, and optical microscopy. Translation remains restricted by bulky hardware, slow acquisition, and probe miniaturization challenges [44,45,46].
- **Hyperspectral and Multispectral Imaging (HSI/MSI):** Capture images at multiple wavelengths beyond standard red/green/blue endoscopy. Multispectral imaging (MSI) uses a limited number of discrete bands (≈5–20), while hyperspectral imaging (HSI) collects hundreds of contiguous narrow bands, producing a richer spectral signature that distinguishes subtle tissue differences. Early feasibility studies suggest improved detection of gastric and esophageal neoplasia, especially when paired with AI-based classification. Both remain experimental, limited by bulky equipment, slow data acquisition, and lack of large-scale validation [47,48,49,50].
- **Multiphoton Microscopy:** Uses nonlinear femtosecond lasers to achieve label-free, high-resolution imaging of subcellular structures via intrinsic autofluorescence and second-harmonic generation. Ex vivo studies of colorectal lesions reported ~90% sensitivity and specificity, outperforming CLE in diagnostic accuracy. Despite this potential, adoption is restricted by cost, complex laser systems, and lack of miniaturized endoscopes, though prototype devices show feasibility for future translation [51,52].
- **Diffuse Reflectance Spectroscopy (DRS):** Measures wavelength-dependent tissue scattering and absorption to assess composition and vascularity. Pilot studies reported accuracies above 85% for distinguishing neoplastic from non-neoplastic tissue in Barrett’s esophagus and colonic polyps. It is inexpensive and less prone to motion artifacts, but remains limited by point-based sampling without spatial mapping, which reduces practicality in real-time endoscopy [53,54].

### 3.7. Magnification Endoscopy

Magnification endoscopy provides optical zoom (up to ~150×) for detailed visualization of mucosal pit and vascular patterns, often combined with chromoendoscopy or virtual chromoendoscopy (e.g., NBI). It improves detection and characterization of early gastric cancer and colorectal neoplasia. A randomized trial demonstrated that magnifying optical enhancement significantly outperformed WLE for detecting gastric intestinal metaplasia and neoplasia [55]. Similarly, a meta-analysis of colorectal lesions reported pooled sensitivity of 89% and specificity of 85.7% for Kudo’s pit pattern classification using magnification [56]. Frameworks such as the vessel-plus-surface (VS) classification and MESDA-G algorithm are widely validated for early gastric cancer diagnosis [14]. Magnification endoscopy is increasingly guideline-supported in gastric and colorectal settings, but remains limited by specialized equipment and longer procedure times.

### 3.8. Integration vs. Probe-Based Systems

Optical imaging technologies differ not only in mechanism but also in how they are deployed during endoscopy. Modalities such as NBI (Olympus), FICE (Fujifilm), i-SCAN (Pentax), AFI, and magnification endoscopy are integrated into endoscope platforms or processors, allowing seamless switching during routine procedures. In contrast, technologies like confocal laser endomicroscopy (CLE, probe-based Cellvizio), optical coherence tomography (OCT/VLE), Raman spectroscopy, photoacoustic imaging, and diffuse reflectance spectroscopy require dedicated probes passed through the working channel or over-the-wire systems. While probe-based methods can provide near-histologic or depth-resolved information, they increase procedural complexity, cost, and training demands, limiting their use largely to specialized centers.

In summary, these emerging modalities highlight the innovation frontier in GI endoscopy. While early studies demonstrate impressive diagnostic performance, their translation to clinical practice remains constrained by cost, technical complexity, and the absence of large-scale validation.

## 4. Clinical Applications

Given the breadth of potential uses, we focus the discussion on key clinical indications, including Barrett’s esophagus, gastric premalignancy, inflammatory bowel disease, colorectal neoplasia, small bowel disorders, and pancreaticobiliary disease, where optical imaging has most significantly impacted practice.

### 4.1. Barrett’s Esophagus

Barrett’s esophagus (BE) is the main precursor of esophageal adenocarcinoma, and early detection of dysplasia is critical. The current standard, the Seattle protocol of four-quadrant biopsies, is limited by sampling error, inefficiency, and poor adherence, prompting evaluation of advanced imaging to improve surveillance [57,58].

Narrow-band imaging (NBI) has the most robust evidence base. By enhancing mucosal and vascular detail, it enables targeted biopsies with high accuracy. In a prospective tandem endoscopy study of 123 patients, NBI detected dysplasia in 57% of patients compared to 43% using WLE with random biopsies, while requiring fewer samples (mean 4.7 vs. 8.5 per patient) [59]. An international randomized crossover trial of 123 patients across six centers similarly showed that NBI achieved comparable detection of intestinal metaplasia to the Seattle protocol but required significantly fewer biopsies (3.6 vs. 7.6 per patient) and yielded more dysplasia (30% vs. 21%) [12]. A meta-analysis of eight studies including 835 patients reported pooled sensitivity of 91% and specificity of 85% for dysplasia detection with NBI [60]. The ASGE PIVI meta-analysis further confirmed that NBI meets the performance thresholds (≥90% sensitivity, ≥80% specificity, and ≥98% negative predictive value) required to replace random biopsies in BE surveillance [61]. Complementing this, a systematic review and meta-analysis of advanced imaging technologies found that they increase dysplasia/neoplasia detection in BE by 34% compared with standard WLE and random biopsies [62]. Collectively, these data underpin ASGE and ESGE endorsements of NBI as an alternative to random biopsies for Barrett’s surveillance [16,63]. However, guidelines emphasize that, in routine practice, particularly outside expert centers, targeted imaging is best combined with random biopsies to minimize the risk of missed dysplasia.

Acetic acid chromoendoscopy is another validated approach for BE surveillance. By transiently acetylating cellular proteins, topical acetic acid induces a whitening effect that quickly fades back to pink, enhancing mucosal contrast and highlighting neoplastic areas for targeted biopsies. The multicenter ABBA randomized crossover trial demonstrated that acetic-acid–targeted biopsies (Portsmouth protocol) reduced biopsy numbers almost ten-fold compared with the Seattle protocol while maintaining detection of high-grade dysplasia and cancer [64]. Routine use, however, has been limited by the additional procedure time (typically 2–8 min), a learning curve for interpretation, and lack of reimbursement. The ESGE position statement recognizes acetic acid chromoendoscopy as a validated option in expert centers with high Barrett’s volumes, but does not recommend it for widespread surveillance [65]. In practice, it is most useful in high-risk or referral populations, or in centers already proficient in advanced chromoendoscopy.

Confocal Laser Endomicroscopy (CLE) provides near-histologic, in vivo imaging, but trial results have been mixed. A single-center RCT (*n* = 101) showed higher neoplasia detection than the Seattle protocol (28 vs. 13 lesions) and reduced the need for random biopsies, whereas a multicenter RCT (*n* = 129) in post-ablation patients found no added benefit over HD-WLE (4.3% vs. 4.7%) [66,67]. An international RCT (*n* = 192) reported more favorable results, with CLE tripling neoplasia detection (22% vs. 6%) and reducing biopsy burden five-fold [68]. Despite these data, its adoption remains limited by cost, narrow field of view, fluorescein use, and lack of reimbursement, and it is not guideline-endorsed for routine BE surveillance.

Other modalities remain investigational. Optical coherence tomography (OCT/VLE) enables subsurface imaging and detection of buried Barrett’s glands after ablation, but uptake is limited by cost, workflow complexity, and need for expert interpretation [69,70,71,72]. Autofluorescence imaging (AFI) initially showed promise but adds little incremental yield (~2%) and carries high false-positive rates (70–80%) in multicenter studies [22,73]. Fluorescence molecular imaging (FMI) with targeted probes (e.g., lectins, EGFR antibodies) has shown feasibility in pilot studies, yet remains experimental due to regulatory, safety, and logistical hurdles [74,75].

From a patient-centered perspective, the key impact of optical imaging in BE is its ability to reduce the number of random biopsies while maintaining or improving dysplasia detection. NBI and CLE both meet this benchmark, while AFI increases unnecessary biopsies due to poor specificity. OCT and CLE can add staging value by visualizing subsurface or cellular-level abnormalities. Importantly, dye-based chromoendoscopy with acetic acid remains highly sensitive, but requires additional procedure time (2–8 min), limiting routine adoption compared with faster virtual techniques.

Clinical takeaway: NBI is the most validated and guideline-endorsed modality for BE surveillance, reducing biopsy burden while maintaining high detection. Acetic acid chromoendoscopy is effective in expert centers but limited by procedure time. CLE and OCT/VLE provide advanced diagnostic insights but remain confined to specialized practice. AFI is obsolete, while FMI is still in early investigational stages (Table 2).

### 4.2. Gastric Premalignant Lesions and Early Gastric Cancer

#### 4.2.1. Gastric Intestinal Metaplasia (GIM)

GIM is a recognized premalignant condition in the Correa cascade toward gastric adenocarcinoma. Accurate detection and mapping are essential for risk stratification and surveillance, yet conventional white-light endoscopy (WLE) often underestimates GIM, especially when lesions are flat or patchy, leading to inefficient and error-prone random biopsies [76].

NBI with magnification is the most validated modality for GIM detection. In a multicenter Japanese feasibility study of 242 patients, magnifying NBI achieved sensitivity of 87% and specificity of 97% for GIM, with overall diagnostic accuracy of 96% [77]. A large cohort study (>2000 patients) reported diagnostic accuracies exceeding 90% for both GIM and early gastric cancer [55]. In a randomized trial of 232 patients, optical enhancement with magnification significantly outperformed WLE for detecting GIM and neoplasia (accuracy 88% vs. 62%, *p* < 0.01) [78]. A 2023 meta-analysis of 16 studies including 2953 patients confirmed pooled sensitivity of 87% and specificity of 86% across 16 studies [79]. Collectively, these data establish NBI with magnification as the first-line tool for GIM surveillance and underpin its endorsement in ESGE and Asian guidelines [55,77,78,79,80].

CLE provides near-histologic, in vivo imaging. In a randomized trial of 122 patients, Li et al. reported per-biopsy GIM detection of 65.7% with CLE vs. 15.7% with WLE, reducing biopsy burden by ~68% [81]. Zuo et al. confirmed higher accuracy with CLE + FICE (75% vs. 31.5% with WLE), and a meta-analysis of seven studies including 450 patients showed pooled sensitivity of 97% and specificity of 94% [76,82]. Despite these strengths, CLE is limited to expert centers due to cost, fluorescein use, and workflow complexity.

Adjunctive methods such as acetic acid chromoendoscopy, FICE, and i-SCAN offer incremental benefits, particularly in combination with CLE, but their standalone performance is weaker than NBI and adoption remains limited [76,80]. Experimental approaches (OCT, AFI, Raman spectroscopy) show promise in early studies, but lack clinical validation for routine GIM surveillance.

Patient-centered outcomes are central. Both NBI and CLE reduce random biopsies, lowering discomfort, cost, and pathology workload. In RCTs, CLE reduced biopsy numbers by two-thirds while maintaining yield, and NBI demonstrated similar efficiency gains [55,78,81]. Meta-analyses confirm NBI achieves high diagnostic accuracy with fewer biopsies [80]. CLE provides real-time histology-like assessments that can accelerate diagnosis-to-treatment, but procedure time and accessibility limit broader uptake.

Clinical takeaway: NBI with magnification is the most practical and guideline-endorsed method for GIM detection, while CLE delivers unmatched accuracy and biopsy reduction in expert centers. Acetic acid, FICE, and i-SCAN provide modest incremental benefit, and investigational modalities remain unproven (Table 3).

#### 4.2.2. Early Gastric Cancer (EGC)

EGC is a pivotal stage where endoscopic resection (EMR/ESD) can be curative. Accurate detection and precise margin delineation are key, but WLE often misses subtle lesions.

NBI with magnification is the cornerstone for EGC diagnosis. By enhancing surface and vascular architecture, it enables classification systems such as the VS (vascular surface) and MESDA-G algorithms. Large multicenter studies report diagnostic accuracy of 98%, with sensitivity of 86% and specificity of 99% [77]. Matsumoto et al. confirmed high accuracy but highlighted reduced sensitivity in fundic gland-type cancers [83]. Meta-analyses show pooled sensitivity and specificity of ~90%, forming the evidence base for ESGE and Japanese guideline endorsement [80,81].

Acetic acid chromoendoscopy (±indigo carmine) remains the most validated method for margin delineation. RCTs confirm superior delineation accuracy. In a study of 92 patients (98 EGCs), Lee et al. demonstrated high accuracy for demarcating differentiated cancers [84]. Hong et al. extended these findings in 205 patients (215 EGCs), showing that mucin phenotype affects border detection [85]. Numata et al., in a prospective series of 63 patients, showed that it significantly reduced positive lateral margins after ESD [86]. While highly sensitive, it adds 2–8 min to procedure time and requires expertise, limiting widespread use.

Adjunctive modalities such as CLE can provide real-time histology-like confirmation of neoplasia, but their role in margin delineation is limited [81]. OCT and AFI remain investigational in EGC.

Patient-centered outcomes include fewer unnecessary biopsies, faster diagnostic pathways, and more precise resections. NBI provides real-time characterization that can obviate random biopsies, while acetic acid chromoendoscopy reduces incomplete resections, thereby lowering recurrence and the need for repeat procedures [86].

Clinical takeaway: NBI is guideline-endorsed and highly accurate for EGC detection, while acetic acid chromoendoscopy is best validated for pre-resection margin delineation. CLE plays a niche role, and OCT/AFI remain investigational. Collectively, these modalities improve curative outcomes through more precise detection and staging (Table 4).

### 4.3. Inflammatory Bowel Disease

Patients with longstanding ulcerative colitis (UC) or Crohn’s colitis carry an elevated colorectal cancer risk, making high-quality surveillance essential. High-definition white-light endoscopy (HD-WLE) with random four-quadrant biopsies has been the traditional approach, but is limited by sampling error, inefficiency, and poor adherence, especially for flat/inapparent dysplasia [87,88,89].

Dye-spray chromoendoscopy (CE) with indigo carmine or methylene blue improves mucosal contrast and enables targeted biopsies. In a landmark RCT of 165 UC patients, Kiesslich et al. detected 32 neoplastic lesions with CE vs. 10 with WLE (*p* < 0.001) while requiring fewer biopsies [90]. Meta-analyses of six RCTs including 1277 patients confirm a 2–3-fold higher dysplasia detection with CE compared with WLE (pooled RR ≈ 2.0) [91]. In a multicenter RCT of 188 patients across 11 centers, Bisschops et al. found CE superior to NBI, particularly for flat lesions [92]. These data underpin SCENIC recommendations that CE with targeted biopsies is the preferred surveillance strategy, with ESGE issuing similar guidance [87,93]. Limitations include an added ~10–15 min for dye application and inspection plus a training requirement, which reduce uptake despite clear efficacy [87,89,94].

Virtual chromoendoscopy (NBI, FICE, i-SCAN) digitally enhances mucosal detail without dye. Early RCTs showed equivalence to HD-WLE but no superiority [95,96]. The FIND-UC trial including a multicenter RCT of 210 patients confirmed CE remained superior to trimodal imaging (HD-WLE + NBI + AFI) for dysplasia yield [97,98]. Syntheses conclude that HD dye CE is the only modality consistently superior to HD-WLE, while HD-NBI and other virtual methods are generally equivalent to HD-WLE but inferior to CE for flat lesions [99,100]. Guidelines permit HD-WLE or virtual CE when dye-based CE is impractical [87,93]. Virtual methods are faster, simpler, and dye-free, reducing reliance on random biopsies compared with legacy WLE, but typically do not exceed CE in detection [89,99,100].

CLE can provide in-vivo microscopic assessment and reduce random biopsies in expert hands, but cost, fluorescein, a narrow field of view, and added time restrict adoption [101,102]. AFI has high false-positive rates and is not recommended for routine surveillance [95,98]. OCT, Raman and other advanced optics remain investigational for IBD surveillance [101,103].

For Crohn’s colitis, evidence is sparser and more heterogeneous than in UC because of segmental disease and strictures, which make dye application and dysplasia recognition more challenging. Small series and expert consensus recommend applying the same surveillance principles used in UC (prefer CE when feasible; HD-WLE/virtual otherwise) to Crohn’s colitis with extensive colonic involvement [87,89,104].

Patient-centered outcomes: CE increases dysplasia yield and reduces unnecessary random biopsies, but prolongs procedures by ~10–15 min and requires expertise [87,89,94]. Virtual CE offers faster, dye-free alternatives that broaden access but usually perform similarly to HD-WLE and below dye CE [98,99,100]. CLE may streamline decision-making in expert centers, but adds cost and time [101,102].

Clinical takeaway: For IBD surveillance, dye-spray CE is the reference standard and guideline-endorsed for highest dysplasia yield. Virtual CE and HD-WLE are acceptable, practical alternatives when dye CE is not feasible. AFI is obsolete, CLE is niche, and other optics remain investigational. In Crohn’s colitis, surveillance practices largely extrapolate from UC. Overall, optical imaging has shifted IBD surveillance toward targeted, efficient, and patient-centered strategies (Table 5).

### 4.4. Colorectal Polyps and Neoplasia

Colorectal cancer (CRC) develops along the adenoma-carcinoma sequence, and adenoma detection rate (ADR) is a key quality indicator inversely correlated with interval cancer risk [105,106]. Conventional WLE misses a proportion of flat or serrated polyps, motivating the use of optical imaging to enhance detection, characterization, and risk stratification.

NBI is the most extensively validated virtual chromoendoscopy technique for polyp detection and real-time optical diagnosis. By enhancing mucosal and vascular detail, NBI supports classification systems such as the NICE and JNET, enabling endoscopists to distinguish adenomas, hyperplastic polyps, and invasive cancers [107,108,109]. Validation studies demonstrate high diagnostic accuracy: a meta-analysis of 28 studies reported pooled sensitivity of 93% and specificity of 83% for neoplastic polyps; Patrun et al. showed accuracy of 89% in community practice [110,111]. While performance can vary, particularly for sessile serrated lesions (SSLs) and in non-expert settings, the WASP system has improved SSL recognition [112]. The DISCARD 2 trial highlighted real-world limitations, finding that NBI fell below the ASGE PIVI thresholds outside expert centers. In expert hands, however, sensitivity and specificity typically exceed 90%, and NBI meets the PIVI criteria for “resect-and-discard” (diminutive adenomas) and “diagnose-and-leave” (rectosigmoid hyperplastic polyps) strategies [107,108,109].

Dye-based CE with indigo carmine or methylene blue significantly increases ADR and flat lesion detection. In a landmark multicenter RCT of 660 patients, Pohl et al. reported an 11% increase in ADR with CE compared with HD-WLE (36.3% vs. 25.3%) [105]. Similarly, Stoffel et al. showed CE identified more flat adenomas and serrated lesions than intensive WLE. Meta-analyses confirm CE’s superiority in high-risk settings, particularly for flat and serrated lesions [113]. However, CE typically adds 10–15 min per procedure and requires training in dye application, limiting its uptake beyond tertiary centers [114].

CLE enables near-histologic evaluation of pit and vascular patterns. Prospective trials show diagnostic accuracies >90%, and meta-analysis reports pooled sensitivity of 81% and specificity of 88% [115,116,117]. Endocytoscopy (EC) achieves ultra-high magnification with topical stains, with accuracies exceeding 95% in expert series. A 2023 meta-analysis of AI-assisted EC reported pooled accuracy of 93% and sensitivity of 94%, supporting its feasibility for optical biopsy [118]. Despite high performance, both modalities are limited by cost, workflow complexity, and availability.

Newer image-enhancement technologies show promise. Linked color imaging (LCI) increased ADR compared with HD-WLE (58.7% vs. 46.7%) in a multicenter RCT [119]. AI-assisted colonoscopy significantly improves ADR without prolonging procedure time, as demonstrated by Park et al. and Liu et al. [120,121]. OCT with deep learning has shown feasibility for in vivo differentiation of benign, adenomatous, and invasive lesions [122]. While encouraging, these modalities remain largely investigational and await broader validation.

For established or advanced neoplasia, optical imaging is used to assess margins, delineate invasion depth, and guide resection strategies. Magnifying NBI and the JNET classification allow discrimination of superficial vs. deeply invasive cancer, guiding EMR vs. surgical referral [108,109]. CLE and EC provide microscopic confirmation of neoplasia, while OCT offers subsurface evaluation of invasion depth. These modalities enhance staging precision but remain confined to expert centers due to equipment cost, training, and workflow considerations.

Patient centered outcomes: NBI enables resect-and-discard and diagnose-and-leave strategies, reducing reliance on histopathology and lowering costs [107,108,109,123]. CE improves ADR and flat/serrated detection but increases procedure time and requires dye expertise [105,113,114]. CLE/EC reduce biopsy burden and may expedite decision-making in expert centers, though limited by accessibility [105,106,107,108,109,110,111,112,113,114,115,116,117,118]. AI-assisted colonoscopy increases ADR without prolonging procedures, supporting broader adoption [120,121]. Importantly, higher ADRs achieved through optical enhancement correlate with reduced interval cancer incidence and mortality [106].

Clinical takeaway: NBI, supported by the NICE and JNET classifications, is the most practical and guideline-endorsed tool for routine optical diagnosis of colorectal polyps. Dye-based CE remains the most sensitive method for flat and serrated lesions, but is constrained by time and training requirements. CLE and EC deliver near-histologic accuracy, with growing evidence for AI augmentation, but are limited to expert centers. Emerging tools such as LCI, OCT, and AI-assisted systems show promise in further improving ADR and diagnostic precision. Together, these modalities move surveillance toward a more accurate, cost-effective, and patient-centered approach to CRC prevention (Table 6).

### 4.5. Small Bowel Disorders

Capsule endoscopy (CE) is the standard non-invasive modality for small bowel evaluation in obscure gastrointestinal bleeding, suspected Crohn’s disease, and small bowel tumors [124]. Its strengths include complete small bowel visualization and high patient tolerability, though subtle vascular lesions and small ulcers may still be missed due to reader fatigue [125].

Virtual chromoendoscopy modes such as Fuji Intelligent Color Enhancement (FICE), Blue Mode, and i-SCAN have been integrated into capsule platforms. Imagawa et al. reported that CE-FICE detected 48 vs. 17 angioectasias compared with standard CE (*p* < 0.001) without prolonging reading time [125]. Krystallis et al. found that Blue Mode improved vascular lesion visibility in 82% of cases, though FICE sometimes worsened overall image quality [126]. Cotter et al. confirmed that optimized FICE settings enhanced delineation of angioectasias, ulcers, and villous edema with strong interobserver agreement [124]. Collectively, virtual CE modes improve recognition of vascular and inflammatory changes, but tumor yield is modest [127].

In device-assisted enteroscopy (DAE), dye chromoendoscopy and virtual enhancement (i-SCAN, NBI) have been tested for subtle small bowel lesions. Small series suggest improved delineation of angioectasias and ulcers, especially in obscure bleeding, but the evidence base remains limited compared with capsule studies.

Beyond software filters, intelligent chromo capsule endoscopy (ICCE) integrates optical enhancement into capsule hardware. In a prospective evaluation, ICCE achieved sensitivity of 95.8% and specificity of 91.7% for early GI tumors, outperforming standard CE (78%/77%) [128]. While early, this underscores the potential of next-generation capsules to improve diagnostic confidence without dyes.

AI-assisted capsule reading is rapidly advancing. A 2025 meta-analysis reported pooled sensitivity of 94%, specificity of 97%, and AUC of 0.99 for Crohn’s disease, with markedly faster reading times than manual review [127]. Algorithms have also been validated for ulcers, vascular lesions, and strictures, with exploratory work in bleeding localization and tumor recognition. However, most rely on small, single-center datasets, and multicenter external validation remains limited.

Clinical perspective: Virtual enhancement modes reduce missed vascular lesions without added risk or cost, though interpretation variability persists. ICCE and AI show potential to enhance accuracy and efficiency, with AI particularly promising for standardizing reads and shortening reporting times [124,125,126,127,128,129].

Clinical takeaway: CE remains the standard for small bowel evaluation. FICE and Blue Mode improve vascular lesion visibility, ICCE shows promise for early tumor detection, and AI offers superior efficiency and diagnostic consistency, though both remain investigational.

### 4.6. Pancreaticobiliary Disorders

Indeterminate biliary strictures and pancreatic cystic lesions remain among the most difficult problems in GI endoscopy. Conventional ERCP sampling has sensitivity below 50%, prompting the adoption of advanced visualization and optical imaging to improve diagnostic accuracy [130,131].

Digital single-operator cholangioscopy (DSOC, SpyGlass DS) is now the reference platform. Although not an optical imaging technique per se, it enables high-definition intraductal visualization and targeted biopsies. A 2025 meta-analysis reported pooled sensitivity of 94% and specificity of 95% for visual impression, while DSOC-guided biopsies achieved ~74% sensitivity and >95% specificity. These data support the 2023 ASGE guideline recommending DSOC as first-line for strictures of undetermined etiology [132,133,134].

Probe-based confocal laser endomicroscopy (pCLE) provides in vivo, near-histologic imaging of biliary epithelium after intravenous fluorescein injection. Initial multicenter registries using the Miami Classification demonstrated very high sensitivity, approaching 98%, but had specificity as low as 67% because inflammatory changes often mimicked malignancy. The Paris Classification refined the diagnostic framework by adding criteria for benign inflammatory strictures, improving specificity to about 80%. Despite these advances, pCLE adoption has remained limited due to high cost, need for fluorescein, interobserver variability, and the modest incremental benefit it offers over DSOC with targeted biopsies. As a result, it is largely restricted to expert centers and has not been incorporated into guidelines [130,131,135].

Optical coherence tomography (OCT) generates cross-sectional images of the biliary wall with ~10 μm resolution. Small studies suggest it can differentiate benign from malignant strictures, but large-scale validation is lacking, and the technology remains investigational [136].

EUS-guided needle-based CLE (nCLE) has shown promise in pancreatic cysts, especially IPMNs. Recent studies report sensitivity and specificity above 85–90% for cyst subtype and dysplasia detection, with patterns correlating to histology [134,137]. nCLE may help avoid unnecessary surgery for low-risk cysts and enable earlier detection of high-risk lesions. Pilot work with AI-assisted interpretation has further improved accuracy and reproducibility [134].

Clinical takeaway: In clinical practice, DSOC has become the standard for indeterminate biliary strictures, while pCLE and OCT remain adjunctive and investigational. For pancreatic cysts, nCLE represents one of the most promising innovations, but is still confined to expert centers. Overall, optical imaging in the pancreaticobiliary tract is advancing toward more accurate, less invasive diagnosis, but only DSOC is established in routine care.

## 5. Summary

This review set out to clarify which optical imaging modalities are guideline-endorsed and suitable for clinical practice, how comparative evidence supports their diagnostic accuracy and cost-effectiveness, and where emerging technologies currently stand. Across indications, narrow-band imaging and dye-based chromoendoscopy consistently met evidence thresholds and are guideline-recommended, while modalities such as CLE, OCT, AFI, Raman spectroscopy, and fluorescence molecular imaging remain investigational with limited clinical translation. By framing optical imaging in terms of evidence readiness and barriers to adoption, we provide an updated, clinically relevant synthesis that aligns with the research questions outlined in the introduction.

## 6. Conclusions and Future Directions

Optical imaging technologies have expanded the diagnostic capabilities of gastrointestinal endoscopy far beyond conventional white-light evaluation. Among them, narrow-band imaging and dye-spray chromoendoscopy have the most robust evidence base and are guideline-endorsed in specific contexts such as Barrett’s esophagus, inflammatory bowel disease, and colorectal neoplasia. Acetic acid chromoendoscopy, particularly in Barrett’s surveillance and early gastric cancer, provides additional value in expert hands. By contrast, modalities such as confocal laser endomicroscopy, optical coherence tomography, and fluorescence molecular imaging offer unique insights but remain confined to specialized or investigational settings due to cost, complexity, and limited validation.

For practicing endoscopists, the key implication is that while certain optical imaging tools are already integrated into guidelines and can meaningfully enhance detection and characterization, others are best viewed as adjunctive or investigational. Recognizing this evidence hierarchy is critical to balancing innovation with practical implementation.

Future progress in optical imaging will likely center on bridging the gap between innovation and routine adoption. Large, multicenter trials are expected to play a key role in validating effectiveness, cost-efficiency, and reproducibility across diverse clinical settings. Investigational technologies such as fluorescence molecular imaging, intelligent chromo capsule endoscopy, and novel spectroscopic approaches will require streamlined regulatory approval and standardization before clinical translation. Artificial intelligence, already integrated into endoscopy for polyp detection and capsule reading, is anticipated to further enhance optical imaging by reducing interobserver variability and improving efficiency. Looking ahead, the development of multimodal platforms that integrate surface, subsurface, and molecular information may usher in a new era of precision endoscopy, informing not only detection but also risk stratification and therapeutic decision-making.

## Figures and Tables

**Table 1:** Classification of optical imaging modalities in GI endoscopy.

Modality	Principle	Manufacturer/Platform	Clinical Role	Evidence Level	Key Limitations
White-Light Endoscopy (WLE)	Broad-spectrum visible light illumination	Universal	Baseline endoscopic imaging for all GI disorders	Gold standard, foundation	Limited sensitivity for subtle/flat lesions, no subsurface detail
Narrow-Band Imaging (NBI)	Optical filters (blue/green narrow bands) accentuate hemoglobin absorption	Olympus	Barrett’s surveillance, early gastric cancer, colon polyp detection	Strong RCTs and guideline-endorsed	Platform-specific; learning curve
FICE (Fuji Intelligent Color Enhancement)	Digital spectral post-processing	Fujifilm	Colon polyp characterization, gastric lesion assessment	Moderate; smaller clinical studies	Less validated vs. NBI; platform-specific
i-SCAN	Digital image post-processing (surface, contrast, tone enhancement)	Pentax	Barrett’s esophagus, IBD assessment, colon polyps	Moderate; clinical feasibility data	Limited RCT evidence; adoption tied to platform
Autofluorescence Imaging (AFI)	Excites intrinsic mucosal fluorophores	Olympus (integrated in some systems)	Early neoplasia detection (Barrett’s, gastric, lung)	High sensitivity but poor specificity	False positives (40–80%), reduced adoption
Fluorescence Molecular Imaging (FMI)	Exogenous fluorescent probes (antibodies, peptides)	Experimental	Targeted dysplasia/neoplasia detection	Early-phase, pilot human studies	Regulatory barriers, probe approval
Confocal Laser Endomicroscopy (CLE)	Laser scanning confocal optics for cellular imaging	Mauna Kea (Cellvizio)	Optical biopsy in Barrett’s, gastric IM, IBD surveillance	RCTs show high accuracy	Cost, training, limited availability
Endocytoscopy	Ultra-high magnification endoscopy	Olympus (prototype)	In vivo nuclear/cellular visualization	Early clinical studies	Niche use, training-intensive
Optical Coherence Tomography (OCT/VLE)	Low-coherence interferometry, cross-sectional “optical ultrasound”	NinePoint, others	Barrett’s staging, biliary/pancreatic strictures	Feasibility and pilot studies	Complex workflow, niche adoption
Raman Spectroscopy	Inelastic light scattering → molecular fingerprint	Experimental	Differentiation of benign vs. malignant lesions	Pilot studies	Weak signal, technical complexity
Photoacoustic Imaging (PAI)	Laser-induced ultrasound emission	Experimental	Functional and structural GI imaging	Preclinical/early clinical	Limited translation, device miniaturization
Hyperspectral/Multispectral Imaging	Captures wide/multiple wavelength bands	Experimental	Tissue “spectral mapping” for neoplasia detection	Pilot human studies	Still experimental, workflow issues
Multiphoton Microscopy	Two-photon excitation, deep 3D imaging	Experimental	High-resolution “optical biopsy”	Preclinical studies	Not miniaturized, limited to labs

**Table 2:** Comparison of optical imaging modalities in BE surveillance.

Modality	Performance	Guideline Status	Advantages	Limitations
NBI	Sensitivity ~91%, specificity ~85%; fewer biopsies (3.6 vs. 7.6); higher dysplasia yield (30% vs. 21%)	ASGE and ESGE endorsed as alternative to Seattle in expert centers	Most validated; reduces biopsy burden, improves dysplasia detection	Requires expertise; guidelines still recommend combining with random biopsies outside expert centers
Acetic Acid CE	~10-fold fewer biopsies; maintained HGD/CA detection	ESGE validated in expert centers; not routine	Inexpensive; safe; effective for dysplasia detection	Extra procedure time (2–8 min); learning curve; not widely adopted
CLE	Mixed RCT results; sensitivity up to 96%; some trials negative	Not guideline-endorsed	Provides real-time, high-resolution imaging	Costly; fluorescein required; limited availability; narrow field of view
OCT/VLE	Detects buried glands; sensitivity ~92%, low specificity	Investigational	Adds subsurface imaging, staging value	High cost; complex workflow; expert interpretation required
AFI	Minimal yield (+2%); false positives 70–80%	Not recommended	Initially promising for neoplasia detection	Very poor specificity; abandoned in practice
FMI	Feasible in pilot studies; targeted probe-based detection	Investigational	Early-stage research only	Potential for molecular targeting

**Table 3:** Optical imaging in gastric intestinal metaplasia (GIM).

Modality	Performance	Guideline Status	Advantages	Limitations
NBI (magnification)	Accuracy >90%; pooled sensitivity/specificity ~85–90%	ESGE and Asian guidelines endorsed	First-line, practical tool; widely available; high accuracy	Operator dependent; reduced sensitivity in subtle/patchy GIM
CLE	Sensitivity 97%, specificity 94%; reduced biopsy burden by ~68%	Not guideline-endorsed	Optical biopsy; reduces need for random biopsies	High cost; fluorescein use; limited to expert centers
Acetic Acid/FICE/i-SCAN	Modest incremental accuracy; improves when combined with CLE	Limited evidence	Useful adjuncts in some contexts	Not reliable stand-alone tools; lack widespread validation
OCT, AFI, Raman	Feasibility data only	Investigational	Potential for early-stage research	Not validated; no established clinical application

**Table 4:** Optical imaging in early gastric cancer (EGC).

Modality	Performance (Evidence)	Guideline Status	Advantages	Limitations
NBI (magnification)	Accuracy up to 98%; sensitivity ~86%, specificity ~99%	ESGE and Japanese guidelines endorsed	First-line for detection and characterization	Requires expertise; reduced sensitivity for fundic gland-type cancers
Acetic Acid Chromoendoscopy	Improves margin delineation; reduces positive lateral margins after ESD	ESGE endorsed in expert centers	Gold standard for resection planning	adds 2–8 min; requires training; limited use outside expert centers
CLE	Confirms neoplasia but limited for margin mapping	Not guideline-endorsed	Adjunct for histology-like confirmation	High cost; narrow field; limited availability
OCT/AFI	Limited pilot data	Investigational	Potential research applications	Not validated; no established clinical role

**Table 5:** Optical imaging in IBD surveillance.

Modality	Performance (Evidence)	Guideline Status	Advantages	Limitations
Dye-spray CE (indigo carmine/methylene blue)	2–3x higher dysplasia detection vs. WLE; fewer biopsies	SCENIC and ESGE: Preferred strategy	Reference standard; highest yield	Adds 10–15 min; requires training; limited uptake outside expert centers
Virtual CE (NBI, FICE, i-SCAN)	Equivalent to HD-WLE; inferior to CE for flat lesions; faster and dye-free	SCENIC and ESGE: Acceptable alternative if dye CE impractical	Widely available; practical alternative; reduces random biopsies	Lower sensitivity for flat lesions; generally not superior to dye CE
CLE	Real-time histology-like imaging; sensitivity ~90%,	Not guideline-endorsed	reduces need for random biopsies	High cost; fluorescein use; narrow field; requires expertise
AFI	High false positives (50–80%); minimal incremental yield	Not recommended	Initially explored as adjunct	Abandoned due to poor specificity
OCT, Raman, others	Feasibility only	Investigational	Potential research applications	Not validated; research use only

**Table 6:** Optical Imaging modalities for colorectal polyps and neoplasia detection.

Modality	Performance	Guideline Status	Advantages	Limitations
NBI (NICE/JNET/WASP)	Sensitivity up to 93%, specificity 83%; accuracy ~90% in experts, ~89% in community; WASP improves SSL detection; PIVI met in expert hands	ASGE PIVI thresholds met in expert centers; guideline-endorsed for optical diagnosis	First-line for real-time characterization; supports resect-and-discard and diagnose-and-leave strategies	Performance lower in non-expert settings; less accurate for SSLs
Dye-based CE	ADR ↑ 11% vs. HD-WLE; superior for flat and serrated lesions; adds 10–15 min	ESGE endorsed in high-risk/flat lesion surveillance	Most sensitive for flat/serrated lesions	Extra procedure time; requires training; limited uptake outside tertiary centers
CLE	Diagnostic accuracies >90% in trials; meta-analysis: sens 81%, spec 88%	Not guideline-endorsed	Enables in-vivo histology; reduces random biopsies	High cost, fluorescein required, limited to expert centers
Endocytoscopy (EC)	Accuracy >95% in expert series; AI-assisted meta-analysis: acc 93%, sens 94%	Not guideline-endorsed	Ultra-high magnification; promising for AI-assisted diagnosis	Technically demanding; limited to research/expert use
LCI	ADR 58.7% vs. 46.7% with HD-WLE	Investigational	Emerging tool; may improve ADR	Limited evidence
AI-assisted colonoscopy	Significant ADR gains without added time	Not guideline-endorsed (evidence growing)	Improves ADR efficiently; practical adjunct	Still investigational; generalizability and training data evolving
OCT	Feasible for in vivo invasion depth and lesion differentiation	Investigational	Subsurface staging	Limited availability; confined to expert centers

## Data Availability

Not applicable.
